# Some fixed points of multivalued maps in multiplicative metric spaces

**DOI:** 10.1016/j.heliyon.2022.e12453

**Published:** 2022-12-15

**Authors:** Aminat O. Ige, Hallowed O. Olaoluwa, Johnson O. Olaleru

**Affiliations:** a Department of Mathematics, Lagos State University, Ojo, Nigeria; b Department of Mathematics, University of Lagos, Akoka, Nigeria

**Keywords:** Multiplicative metric spaces, Multivalued maps, α−ϕ contractions, Graph

## Abstract

In this manuscript, new fixed point theorems for multivalued maps, particularly α−ϕ contractions, in complete multiplicative metric spaces are proved. We further prove a fixed point theorem for multivalued mappings on a graph-equipped multiplicative metric space. Our results extend the result of Dosenovic et al. [4], and generalize the result of Ali et al. [2]. We included an example to validate our result.

## 1 Introduction

Multiplicative metric spaces (MMS) were introduced in the year 2008 by Bashirov et al. [3]. The idea was about replacing the triangle inequality in standard metric spaces (SMS) with a multiplicative triangle inequality. Having realized that the concepts of multiplicative derivative (d⁎erivative) and multiplicative integral (i⁎ntegral) are based on ordinary limit operation, they defined a multiplicative limit (l⁎imit) and multiplicative absolute value which led to the definition of multiplicative distance $d^{⁎}(x,y)={\left|\frac{x}{y}\right|}^{⁎}$ on $R_{+}:=(0,\infty )$. Some of the properties satisfied by the multiplicative distance $d^{⁎}$ gave birth to the general concept of multiplicative metrics, defined as alternative to standard metrics. It was noted in [1] that the set of positive real numbers is not complete in the usual topology (induced by the standard metric dist(x,y)=|x−y|) but it is complete for the multiplicative metric $d^{⁎}$. On the other hand, a sequence $\left\{x_{n}\right\}$ of positive real numbers is convergent with respect to *d* if and only if it is convergent with respect to $d^{⁎}$.

Recall that, on a non-empty set S, a distance or metric is a non-negative (real-valued) function dist defined for all pairs of elements in S, and satisfying the following properties for all μ,ν,ω∈S: (i) dist(μ,ν)=0 ⟺μ=ν; (ii) dist(μ,ν)=dist(ν,μ); and (iii) dist(μ,ω)≤dist(μ,ν)+dist(ν,ω) (triangle inequality). Multiplicative metric spaces are defined thus:

Definition 1.1 [3] Let S be a non-empty set. A function $dist^{\times }:S\times S\to [1,\infty )$ is a multiplicative metric (MM) on S if it satisfies for all μ,ν,ω∈S the following:

(i) $dist^{\times }(\mu ,\nu )=1$ ⟺μ=ν;

(ii) $dist^{\times }(\mu ,\nu )=m^{\times }(\nu ,\mu )$;

(iii) $dist^{\times }(\mu ,\omega )\leq dist^{\times }(\mu ,\nu )\cdot dist^{\times }(\nu ,\omega )$ (multiplicative triangle inequality).

The pair $\left(S,dist^{\times }\right)$ is called a Multiplicative Metric Space (MMS).

Example 1.1 [9] The set $R_{+}^{n}$ of all *n*-tuples of positive real numbers and let $d_{n}^{⁎}:R_{+}^{n}\times R_{+}^{n}\to [1,\infty )$ be a function defined as

$$d_{n}^{⁎}(\mu ,\nu )=\prod_{r=1}^{n}{\left|\frac{\mu_{r}}{\nu_{r}}\right|}^{⁎}$$

where $\mu =(\mu_{1},\mu_{2},\ldots \mu_{n})$, $\nu =(\nu_{1},\nu_{2},\ldots \nu_{n})\in R_{+}^{n}$ and $\left|\cdot {|}^{⁎}:(0,\infty )\to [1,\infty \right)$ is defined for all ϵ>0 by $|\epsilon {|}^{⁎}=\epsilon \chi_{\left[1,\infty \right)}+\frac{1}{\epsilon }\chi_{\left(0,1\right]}$ (*χ* denoting a characteristic function). $d_{n}^{⁎}$ is a multiplicative metric. It is obvious that it is not a standard metric.

In 2012, Ozavsar and Cevikel [9] investigated some topological properties of MMS and proved certain fixed point theorems for contraction mappings of multiplicative metric spaces. The definition that follows was given in [9]:

Definition 1.2 [9] A multiplicative contraction is a self map of a multiplicative metric space $\left(S,dist^{\times }\right)$ such that there is a real constant *λ*, with 0≤λ<1, satisfying $dist^{\times }(f(\mu ),f(\nu ))\leq {\left[dist^{\times }(\mu ,\nu )\right]}^{\lambda }$ for each pair (μ,ν) of points in S.

In [4], it was shown that fixed point results in multiplicative metric spaces can be derived from fixed point results in standard metric spaces and vice-versa with the use of logarithm and exponential maps. The results were however limited to single-valued maps.

Let CB(S) be the set of all non-empty, closed and bounded subsets of a metric space (S,d), and CL(S) the set of all closed subsets of S. The Hausdorff metric induced by the metric dist is the function H:CB(S)×CB(S)→[0,∞) defined by:

$$H(\Delta ,\Gamma )=\max\left\{\underset{\mu \in \Delta }{\sup}dist(\mu ,\Gamma ),\;\underset{\nu \in \Gamma }{\sup}dist(\nu ,\Delta )\right\},$$

where $dist(\mu ,\Gamma )=\underset{\nu \in \Gamma }{\inf}dist(\mu ,\nu )$ and $dist(\nu ,\Delta )=\underset{\mu \in \Delta }{\inf}dist(\mu ,\nu )$.

The concept of multivalued mappings is a widely applied one, and Nadler [8] combined the concept with the notion of contraction maps to establish a well known fixed point theorem on multivalued contraction maps T:S→CB(S), i.e. such that there is λ∈[0,1) satisfying H(Tμ,Tν)≤λdist(μ,ν) for any pair (μ,ν) of points in S, where (S,dist) is a complete metric space.

Samet et al. [10], in 2012, defined the notion of α−ψ contractive mapping as a generalization of contraction maps, and established conditions under which their fixed points exist. In [10], T:S→S is called an α−ψ contraction mapping whenever α(μ,ν)dist(Tμ,Tν)≤ψ(dist(μ,ν)) for all μ,ν∈S, where *α* is a non-negative (real-valued) map defined for all pairs of elements in S, and *ψ* is an increasing self-map of [0,∞) such that for all ϵ>1, the sum $\sum_{n=1}^{\infty }\psi^{n}(\epsilon )$ is finite, $\psi^{n}$ denoting the *n*-th iterate of *ψ*. We denote by Ψ the class of such functions *ψ*.

The notion of α−ψ contractions has been extended to Ćirić-type contractive multivalued maps as defined below:

Definition 1.3 [6], [7] Let (S,dist) be a metric space, α:S×S→[0,∞) a function and ψ:[0,∞)→[0,∞) an increasing map such that the sum $\sum_{n=1}^{\infty }\psi^{n}(\epsilon )$ is finite for any ϵ>1, with $\psi^{n}$ denoting the *n*-th iterate of *ψ*.

A (multivalued) map T:S→CL(S) is called α−ψ contraction of Ćirić type if:

(1) H(Tμ,Tν)≤ψ(M(μ,ν))for μ,ν∈S with α(μ,ν)≥1,

where

(2) $$M(\mu ,\nu )=\max\left\{dist(\mu ,\nu ),\;dist(\mu ,T\mu ),\;dist(\nu ,T\nu ),\;\frac{1}{2}[dist(\mu ,T\nu )+dist(\nu ,T\mu )]\right\}.$$

Note that (1) in Definition 1.3 is equivalent to the condition below, consistent with [10]:

β(μ,ν)H(Tμ,Tν)≤ψ(M(μ,ν))for all μ,ν∈S,

where β:S×S→[0,∞) is the characteristic function $\chi_{\alpha^{-1}([1,\infty ))}$.

In this paper, the existence of fixed points of multivalued α−ψ contractions of Ćirić type in MMS is proved using the equivalence between SMS and MMS. The methodology extends the method used in [4] to multivalued maps. We also discuss some results on fixed points for multivalued mappings when the multiplicative metric space $\left(S,dist^{\times }\right)$ is equipped with a graph. Jachymski [5] used the concept of graph as a replacement of ordering in establishing some fixed point results. There is a striking similarity between contractive maps on spaces equipped with a graph and *α*-*ψ*-contractions. Such similarity will be discussed in this paper as we attempt to extend the main result of Ali et al. [2] in the setting of multiplicative metric spaces equipped with a graph.

## 2 Preliminaries

We begin this section by stating the following result on the equivalence between SMS and MMS.

Proposition 2.1 *[4]* *If* $\left(S,dist^{\times }\right)$ *is a multiplicative metric space, then the pair* (S,dist) *is a metric space, where* dist:S×S→[0,∞) *is defined by* $dist(\mu ,\nu )=\ln\left[dist^{\times }(\mu ,\nu )\right]$ *for all* μ,ν∈S *. Conversely, if* (S,dist) *is a metric space, then* $\left(S,dist^{\times }\right)$ *is an MMS where* $dist^{\times }(\mu ,\nu )=e^{dist(\mu ,\nu )}$ *for all* μ,ν∈S *. Hence,* $\left(S,dist^{\times }\right)$ *is an MMS if and only if* $\left(S,\ln dist^{\times }\right)$ *is an SMS, that is,* (S,dist) *is an SMS if and only if* $\left(S,e^{dist}\right)$ *is an MMS.*

From Proposition 2.1, given that (CB(S),H) is a metric space, $\left(\mathrm{CB}(S),H^{\times }\right)$ is a multiplicative metric space, where $H^{\times }$ is such that $H^{\times }(P,Q)=e^{H(P,Q)}$ for all P,Q∈CB(S), and we write $H^{\times }=e^{H}$ or $H=\ln H^{\times }$. More explicitly, for all P,Q∈CB(S), if $dist(\mu ,Q)=\underset{\nu \in Q}{\inf}dist(\mu ,\nu )$, $dist^{\times }(\mu ,Q)=\underset{\nu \in Q}{\inf}dist^{\times }(\mu ,\nu )$, and $D(P,Q)=\underset{\mu \in P}{\sup}dist(\mu ,Q)$, we have that $e^{D(P,Q)}=\underset{\mu \in P}{\sup}e^{dist(\mu ,Q)}=\underset{\mu \in P}{\sup}dist^{\times }(\mu ,Q)$ so that:

$$\begin{array}{ccc} H^{\times }(P,Q)\; & =\; & \max\left\{e^{D(P,Q)},\;e^{D(Q,P)}\right\}\\ \; & =\; & \max\left\{\underset{\mu \in P}{\sup}dist^{\times }(\mu ,Q),\;\underset{\nu \in Q}{\sup}dist^{\times }(\nu ,P)\right\} \end{array}$$

We thus have the following definition and proposition:

Definition 2.1 Let $\left(S,dist^{\times }\right)$ be a multiplicative metric space and let CB(S) denote the class of non-empty closed and bounded subsets of S. The function $H^{\times }:\mathrm{CB}(S)\times \mathrm{CB}(S)\to [1,\infty )$ defined by:

$$H^{\times }(P,Q)=\max\left\{\underset{\mu \in P}{\sup}dist^{\times }(\mu ,Q),\;\underset{\nu \in Q}{\sup}dist^{\times }(\nu ,P)\right\},\;P,Q\in \mathrm{CB}(S),$$

is the Hausdorff multiplicative metric induced by the multiplicative metric $dist^{\times }$.

Proposition 2.2 *If* (S,dist) *is a metric space and* $\left(S,dist^{\times }\right)$ *is the equivalent multiplicative metric space, where* $dist^{\times }=e^{dist}$ *and* $d=\ln dist^{\times }$ *, then* $H^{\times }$ *is the Hausdorff metric induced by* dist *if and only if* $H^{\times }$ *is the Hausdorff multiplicative metric induced by* $dist^{\times }$ *, where:*

$$H^{\times }(P,Q)=e^{H(P,Q)}\;\text{or}\;H(P,Q)=\ln H^{\times }(P,Q)\;\text{for all }P,Q\text{.}$$

Remark 2.1 The Hausdorff multiplicative metric defined on the space CB(S) of closed and bounded subsets of S can be extended to the collection CL(S) of closed subsets of S by defining the function $H^{⁎}:\mathrm{CL}(S)\times \mathrm{CL}(S)\to [0,\infty )$ by

$$H^{\times }(P,Q)=\left\{ \begin{array}{c} +\infty \text{ if }\max\left\{\underset{\nu \in Q}{\sup}dist^{\times }(\nu ,P),\;\underset{\mu \in P}{\sup}dist^{\times }(\mu ,Q)\right\}\text{ does not exist}\\ \max\left\{\underset{\nu \in Q}{\sup}dist^{\times }(\nu ,P),\;\underset{\mu \in P}{\sup}dist^{\times }(\mu ,Q)\right\}\text{ otherwise}. \end{array}\right.$$

$H^{\times }$ thus defined is called the generalized Hausdorff multiplicative metric and it is clear that in this case, $H=\ln H^{\times }$ (with the convention ln⁡(+∞)=+∞) is the generalized Hausdorff metric and conversely.

The following lemmas and definitions are true in multiplicative metric spaces and are necessary for the sequel:

Lemma 2.1 *[9]* *A sequence* $\left\{u_{n}\right\}$ *in a multiplicative metric space* $\left(S,dist^{\times }\right)$ *is:*

(i) *a multiplicative Cauchy sequence if and only if* $\lim_{p,q\to \infty }dist^{\times }(u_{p},u_{q})=1$ *,*
(ii) *multiplicative convergent to a point* u∈S *if and only if* $\lim_{p\to \infty }dist^{\times }(u_{p},u)=1$ *.*

Lemma 2.2 *[9]* *In multiplicative metric spaces, multiplicative convergent sequences are multiplicative Cauchy sequences.*

Definition 2.2 [9] A multiplicative metric space $\left(S,dist^{\times }\right)$ is said to be complete if multiplicative Cauchy sequences in S converge in S.

Remark 2.2 It then follows from Proposition 2.1, Proposition 2.2 that an MMS $\left(S,dist^{\times }\right)$ is complete if and only if the equivalent SMS (S,dist) is complete. Also, a sequence of points in S is multiplicative convergent (in the MMS $\left(S,dist^{\times }\right)$) to u∈S if and only if it converges to *u* (in the SMS (S,dist)).

Now, we define the concept of α−ϕ multiplicative contraction of Ćirić-type:

Definition 2.3 Let $\left(S,dist^{\times }\right)$ be a multiplicative metric space, let α:S×S→[0,∞) be a map and let ϕ:[1,∞)→[1,∞) an increasing function such that for any ϵ>1, the sum $\sum_{n=1}^{\infty }\ln\phi^{n}(\epsilon )$ is finite, where $\phi^{n}$ denotes the n-th iterate of *ϕ*. Denote by Φ the class of such functions *ϕ*.

A multivalued mapping T:S→CL(S) is called an α−ϕ multiplicative contraction of Ćirić type if for all μ,ν∈S with α(μ,ν)≥1, the following holds:

(3) $$H^{\times }(T\mu ,T\nu )\leq \phi (M^{\times }(\mu ,\nu )),$$

where

(4) $$M^{\times }(\mu ,\nu )=\max\left\{dist^{\times }(\mu ,\nu ),dist^{\times }(\mu ,T\mu ),dist^{\times }(\nu ,T\nu ),\sqrt{dist^{\times }(\mu ,T\nu )\cdot dist^{\times }(\nu ,T\mu )}\right\}.$$

Unlike the space-wide contractive condition of the multivalued contraction maps discussed in [8], the contractive-like condition (3) only holds for pairs (μ,ν)∈S×S such that α(μ,ν)≥1, the function *α* being not necessarily commutative. For fixed point results to be obtained, there's need to evaluate the Hausdorff distance between two terms of an orbit $O_{T}(x)=\{x_{n}:x_{n}\in Tx_{n-1},n\in N\}$ of *T* at a given point x∈S, hence, all pairs $\left(x_{n},x_{n+1}\right)$ of consecutive terms must be such that $\alpha (x_{n},x_{n+1})\geq 1$. The notion of *α*-admissibility defined below thus becomes necessary to serve as transitivity from a pair to another, with the assumption that $\alpha (x_{0},x_{1})\geq 1$.

Definition 2.4 [6] A multivalued function T on a non-empty set S is called *α*-admissible with respect to a map α:S×S→[0,∞) if for each μ∈S and ν∈Tu with α(μ,ν)≥1, we have α(ν,ω)≥1 for all ω∈Tv.

Graph theory somehow replicates the role of the function *α* in the confinement of contractive conditions to a subset of the space S×S. Indeed, let G=(V(G),E(G)) be a directed graph, with V(G) denoting the set of its vertices, and E(G) the set of all its edges. Suppose the following: **(i)** there are no parallel edges in G; **(ii)** V(G) coincides with the points of a multiplicative space $\left(S,dist^{\times }\right)$; and **(iii)** loops are all contained in E(G), i.e. E(G)⊃Δ:={(u,u)|u∈S}. The concept of Banach G-contractions as introduced in [5] has a natural equivalent in the MMS setting. A mapping T:S→S is called a multiplicative Banach G-contraction whenever edges of G are preserved by *T* and their weights are ‘multiplicatively’ decreased by *T*: **(iv)** for all μ,ν∈S, (μ,ν)∈E(G)⇒(Tμ,Tν)∈E(G) and **(v)** for some λ∈(0,1), the inequality $dist^{\times }(T\mu ,T\nu )\leq {\left[dist^{\times }(\mu ,\nu )\right]}^{\lambda }$ holds for all (μ,ν)∈E(G). The graph-contractive condition **(v)** similarly holds for all pairs (μ,ν)∈S×S such that α(μ,ν)≥1, where *α* is the characteristic function $\chi_{E(G)}$.

## 3 Main results

### 3.1 Fixed points of *α*-admissible multivalued maps in MMS

We apply Proposition 2.2 to some existing theorems in the setting of SMS and multivalued mappings. Our results are further presented below as theorems after each classified fixed point theorem for multivalued mappings in SMS. Note that the authors of [4] examined equivalence of results in SMS and MMS in the setting of single-valued maps only.

Theorem 3.1 *[8]**Let*(S,dist)*be a complete metric space and consider a multivalued mapping*T:S→CL(S)*satisfying*H(Tμ,Tν)≤λdist(μ,ν)*for all*μ,ν∈S*, for some real constant*λ∈(0,1)*. The map*T*has then a fixed point*ω∈S*(i.e. such that*ω∈Tω*).*

We deduce from Theorem 3.1 its equivalent in MMS:

Theorem 3.2 *Any multivalued map*T:S→CL(S)*defined on a complete multiplicative metric space*$\left(S,dist^{\times }\right)$*, and such that there is*λ∈(0,1)*satisfying*$H^{\times }(T\mu ,T\nu )\leq {\left[dist^{\times }(\mu ,\nu )\right]}^{\lambda }$*for all pairs*(μ,ν)*of points in*S*, has a fixed point*w∈S*.*

Proof If T:S→CL(S) satisfies the conditions in Theorem 3.2 then,

$$H(T\mu ,T\nu )=\ln H^{\times }(T\mu ,T\nu )\leq \lambda \ln\left[dist^{\times }(\mu ,\nu )\right]=\lambda dist(\mu ,\nu )\;\text{for all }\mu ,\nu \in S,$$

where *H* and dist are as defined in Proposition 2.1, Proposition 2.2. Thus, T satisfies the condition in Theorem 3.1, hence from Theorem 3.1, it has a fixed point. □

Jleli et al. [6] proved the existence of fixed points of α−ψ contractions of Ćirić type on b-metric spaces, under three conditions (see Theorem 14 in [6]). It is worthy of note that a non-empty set is a b-metric space when equipped with a metric-like function $d_{s}$, non-negative, real-valued, defined for all pairs of elements, satisfying the two first properties of a metric and the weakened triangle inequality $d_{s}(\mu ,\omega )\leq s[d_{s}(\mu ,\nu )+d_{s}(\nu ,\omega )]$ for all 3-tuples (μ,ν,ω) of points in the space, where s≥1 is a fixed constant. Thus, a metric space is a b-metric space with s=1. In the following theorem, we write the result of Jleli et al. [6] in the case of metric spaces (i.e. when s=1).

Theorem 3.3 *(see**[6]**) Let*(S,dist)*be a complete metric space and let*T:S→CL(S)*be a multivalued map. If there are functions*α:S×S→[0,+∞)*and*ψ∈Ψ*such that*T*is an*α−ψ*contraction of Ćirić type, then*T*has a fixed point provided all three following conditions hold:*

(i) T*is an α-admissible multivalued mapping;*
(ii) *Some*$\mu_{0}\in S$*and*$\mu_{1}\in Tu_{0}$*satisfy*$\alpha (\mu_{0},\mu_{1})\geq 1$*;*
(iii) *Sequences*$\{\mu_{n}\}\subset S$*convergent to some*μ∈S*, and satisfying*$\alpha (\mu_{n},\mu_{n+1})\geq 1$*for all*n∈N∪{0}*, are such that*$\alpha (\mu_{n},\mu )\geq 1$*for all*n∈N∪{0}*.*

From Theorem 3.3, the following result in MMS is deduced:

Theorem 3.4 *Let*$\left(S,dist^{\times }\right)$*be a complete multiplicative metric space. If for some function*ϕ∈Φ*and some map*α:S×S→[0,∞)*, a multivalued map*T:S→CL(S)*is an*α−ϕ*multiplicative contraction of Ćirić type, then the map*T*admits a fixed point provided all three following conditions hold:*

(a) T*is α-admissible;*
(b) *there exists*$\mu_{0}\in S$*and there exists*$\mu_{1}\in T\mu_{0}$*such that*$\alpha (\mu_{0},\mu_{1})\geq 1$*;*
(c) *given a sequence*${\left\{\mu_{n}\right\}}_{n\geq 0}$*in*S*such that*$\alpha (\mu_{n},\mu_{n+1})\geq 1$*for all*n≥0*and such that*$\mu_{n}\to \mu \in S$*, the inequality*$\alpha (\mu_{n},\mu )\geq 1$*holds for all*n≥0*.*

Proof Take μ,ν∈S be such that α(μ,ν)≥1. The following holds:

$$H(T\mu ,T\nu )=\ln H^{\times }(T\mu ,T\nu )\leq \ln\phi (M^{\times }(\mu ,\nu )).$$

$$\begin{array}{ccc} \phi (M^{\times }(\mu ,\nu ))\; & =\; & \phi \left(\max\left\{\begin{array}{c} dist^{\times }(\mu ,\nu ),\;d^{⁎}(\mu ,T\mu ),\;dist^{\times }(\nu ,T\nu ),\;\sqrt{dist^{\times }(\mu ,T\nu )dist^{\times }(\nu ,T\mu )} \end{array}\right\}\right)\\ \; & =\; & \max\left\{\begin{array}{c} \phi (dist^{\times }(\mu ,\nu )),\;\phi (dist^{\times }(\mu ,T\mu )),\;\phi (dist^{\times }(\nu ,T\nu )),\;\phi \left(\sqrt{dist^{\times }(\mu ,T\nu )dist^{\times }(\nu ,T\mu )}\right) \end{array}\right\}\\ \; & =\; & \max\left\{\begin{array}{c} \phi (e^{dist(\mu ,\nu )}),\;\phi (e^{dist(\mu ,T\nu )}),\;\phi (e^{dist(\nu ,Tv)}),\;\phi \left(\sqrt{e^{dist(\mu ,T\nu )}e^{dist(\nu ,T\mu )}}\right) \end{array}\right\}\\ \; & =\; & \max\left\{\begin{array}{c} \gamma (dist(\mu ,\nu )),\;\gamma (dist(\mu ,T\mu )),\;\gamma (dist(\nu ,T\nu )),\;\gamma \left(\frac{dist(\mu ,T\nu )+dist(\nu ,T\mu )}{2}\right) \end{array}\right\}, \end{array}$$

where $\gamma :[0,\infty )\to [1,\infty ),\;\epsilon \mapsto \gamma (\epsilon )=\phi (e^{\epsilon })$, and where *H* and dist are as defined in Proposition 2.1, Proposition 2.2. Since *γ* is strictly increasing,

$$\begin{array}{ccc} \phi (M^{\times }(\mu ,\nu ))\; & =\; & \gamma \left(\max\left\{\begin{array}{c} dist(\mu ,\nu ),\;dist(\mu ,T\mu ),\;dist(\nu ,T\nu ),\;\frac{1}{2}\left(dist(\mu ,T\nu )+dist(\nu ,T\mu )\right) \end{array}\right\}\right)\\ \; & =\; & \gamma (M(\mu ,\nu )). \end{array}$$

H(Tμ,Tν)≤ln⁡γ(M(μ,ν))=ψ(M(μ,ν)), where ψ:[0,∞)→[0,∞) is defined by ψ(ϵ)=ln⁡γ(ϵ) for all ϵ≥0. *ψ* is strictly increasing,

$$\left\{ \begin{array}{ccccc} \psi (\epsilon )\; & =\; & \ln\gamma (\epsilon )\; & =\; & \ln\phi (e^{\epsilon })\\ \psi^{2}(\epsilon )\; & =\; & \psi (\psi (\epsilon ))\; & =\; & \ln\phi (e^{\psi (\epsilon )})=\ln\phi (\phi (e^{\epsilon }))=\ln\phi^{2}(e^{\epsilon })\\ \; & \vdots \; & \;\\ \psi^{n}(\epsilon )\; & =\; & \ln\phi^{n}(e^{\epsilon }),\; & \; & n\in N, \end{array}\right.$$

and for all $\epsilon >0,\;\sum_{n=1}^{\infty }\psi^{n}(\epsilon )=\sum_{n=1}^{\infty }\ln\phi^{n}(e^{\epsilon })<\infty$.

T is therefore an α−ψ contraction of Ćirić type on the metric space (S,dist), where ψ∈Ψ. When conditions (a) - (c) hold, then, by Theorem 3.3, T has a fixed point, since conditions (i) - (iii) of Theorem 3.3 also hold. □

### 3.2 Fixed point of multivalued maps in MMS and SMS with a graph

In this subsection, we prove some fixed point theorems for multivalued mappings defined on a multiplicative metric space equipped with a graph.

Let $\left(S,dist^{\times }\right)$ be a multiplicative metric space, Δ:={(u,u)|u∈S} the diagonal of S×S, and G=(V(G),E(G)) a directed graph in which there are no parallel edges, and for which the points of S are the vertices of G (i.e. V(G)=S) and loops are edges (i.e. E(G)⊃Δ). By allotting to each edge (μ,ν) the ‘multiplicative distance’ $dist^{\times }(\mu ,\nu )$ between its vertices, G can be viewed as a weighted graph.

Ali et al. [2] discussed some fixed point results for single-valued and multivalued maps defined of b-multiplicative metric spaces equipped with a graph. We state their main theorem in the setting of MMS:

Theorem 3.5 *[2]**Let a multivalued map*T:S→CL(S)*defined on a complete multiplicative metric space*$\left(S,dist^{\times }\right)$*equipped with some graph*G=(V(G),E(G))*, satisfy the following:*

(i) *for each*(μ,ν)∈E(G)*and*ξ∈Tμ*, there exists*η∈Tν*satisfying*
  (5) $$dist^{\times }(\xi ,\eta )\leq {\left[dist^{\times }(\mu ,\nu )\right]}^{\kappa }{\left[dist^{\times }(\nu ,\xi )\right]}^{\omega },\;\;\text{where }\kappa \in [0,1)\text{ and }\omega \geq 0\text{;}$$
(ii) *there exists*$\mu_{0}\in S$*and*$\mu_{1}\in T\mu_{0}$*such that*$(\mu_{0},\mu_{1})\in E$*;*
(iii) *for each*ξ∈Tμ*and*η∈Tν*such that*$dist^{\times }(\xi ,\eta )<dist^{\times }(\mu ,\nu )$*we have*(ξ,η)∈E(G)*whenever*(μ,ν)∈E(G)*;*
(iv) *for each sequence*$\{\mu_{n}\}\subset S$*such that*$\left(\mu_{n},\mu_{n+1})\in E(G\right)$*and*$\mu_{n}\to \mu$*,*$\left(\mu_{n},\mu )\in E(G\right)$*for each*n∈N*.*

*The multivalued mapping*T*has a fixed point.*

We generalize this theorem by weakening the contractive condition (5). To the best of our knowledge, the equivalent in the setting of SMS of the following theorem has not been proved in literature.

Theorem 3.6 *Let*T:S→CL(S)*be a mapping defined on a complete multiplicative metric space*$\left(S,dist^{\times }\right)$*equipped with a graph*G=(V(G),E(G))*, and such that:*

(i) *For any*(μ,ν)∈E(G)*and each*ξ∈Tμ*, there exists*η∈Tν*such that*$dist^{\times }(\xi ,\eta )\leq \phi (M^{\times }(\mu ,\nu ))\cdot f\left(dist^{\times }(\nu ,\xi )\right)$*, where*ϕ∈Φ*,*$M^{\times }$*is as defined in*(4)*, and*f:[1,∞)→[1,∞)*is a continuous function at* 1 *such that* f(1)=1*;*
(ii) *For some*$\mu_{0}\in S$*and*$\mu_{1}\in T\mu_{0}$*,*$\left(\mu_{0},\mu_{1})\in E(G\right)$*;*
(iii) *For any*ξ∈Tμ*and*η∈Tν*such that*$dist^{\times }(\xi ,\eta )<dist^{\times }(\mu ,\nu )$*, the implication*(μ,ν)∈E(G)⇒(ξ,η)∈E(G)*holds;*
(iv) *For each sequence*$\{\mu_{n}\}\subset S$*such that*$\left(\mu_{n},\mu_{n+1})\in E(G\right)$*and*$\mu_{n}\to \mu$*,*$\left(\mu_{n},\mu )\in E(G\right)$*for all*n∈N*.*

*The multivalued mapping*T*has a fixed point.*

Proof Take $\mu_{0}\in S$ and $\mu_{1}\in T\mu_{0}$ be such that $\left(\mu_{0},\mu_{1})\in E(G\right)$. Then from (i), there is $\mu_{2}\in T\mu_{1}$ satisfying

$$\begin{array}{ccc} dist^{\times }(\mu_{1},\mu_{2})\; & \leq \; & \phi (\max\{dist^{\times }(\mu_{0},\mu_{1}),\;dist^{\times }(\mu_{0},\mu_{1}),\;dist^{\times }(\mu_{1},\mu_{2}),\;\sqrt{dist^{\times }(\mu_{0},\mu_{2})\cdot d^{⁎}(\mu_{1},\mu_{1})}\})f(d^{⁎}(\mu_{1},\mu_{1}))\\ \; & =\; & \phi \left(\max\left\{dist^{\times }(\mu_{0},\mu_{1}),\;dist^{\times }(\mu_{1},\mu_{2}),\;\sqrt{dist^{\times }(\mu_{0},\mu_{1})dist^{\times }(\mu_{1},\mu_{2})}\right\}\right)\\ \; & =\; & \phi \left(\max\{dist^{\times }(\mu_{0},\mu_{1}),\;dist^{\times }(\mu_{1},\mu_{2})\}\right). \end{array}$$

If $dist^{\times }(\mu_{0},\mu_{1})<dist^{\times }(\mu_{1},\mu_{2})$, then $dist^{\times }(\mu_{1},\mu_{2})\leq \phi (dist^{\times }(\mu_{1},\mu_{2}))<dist^{\times }(\mu_{1},\mu_{2})$, a clear contradiction. Therefore $dist^{\times }(\mu_{1},\mu_{2})\leq dist^{\times }(\mu_{0},\mu_{1})$ and $dist^{\times }(\mu_{1},\mu_{2})\leq \phi (dist^{\times }(\mu_{0},\mu_{1}))<dist^{\times }(\mu_{0},\mu_{1})$.

From (iii), $\left(\mu_{1},\mu_{2})\in E(G\right)$.

Repeating the process, a sequence $\{\mu_{n}\}\subset S$ is obtained such that $\mu_{n+1}\in T\mu_{n}$, $\left(\mu_{n},\mu_{n+1})\in E(G\right)$ and $dist^{\times }(\mu_{n},\mu_{n+1})\leq \phi (dist^{\times }(\mu_{n-1},\mu_{n}))\leq \ldots \leq \phi^{n}(dist^{\times }(\mu_{0},\mu_{1}))$ for all n∈N. Given natural numbers m,n such that m>n, $dist^{\times }(\mu_{n},\mu_{m})\leq \prod_{k=n}^{m-1}dist^{\times }(\mu_{k},\mu_{k+1})\leq \prod_{k=n}^{m-1}\phi^{n}\left(dist^{\times }(\mu_{0},\mu_{1})\right)=e^{\sum_{k=n}^{m-1}\ln\phi^{n}\left(dist^{\times }(\mu_{0},\mu_{1})\right)}$. Since $\lim_{n\to \infty }\sum_{k=n}^{m-1}\ln\phi^{n}\left(dist^{\times }(\mu_{0},\mu_{1})\right)=0$, it follows that $\lim_{n,m\to \infty }dist^{\times }(\mu_{n},\mu_{m})=0$. Thus $\left\{\mu_{n}\right\}$ is Cauchy hence it converges to some $\mu^{⁎}\in S$.

From (iv), $\left(\mu_{n},\mu^{⁎})\in E(G\right)$ for all n∈N, and from (i), since $\mu_{n+1}\in T\mu_{n}$, there exists $\eta_{n}^{⁎}\in T\mu^{⁎}$ such that:

$$dist^{\times }(\mu_{n+1},\eta_{n}^{⁎})\leq \phi (M^{\times }(\mu_{n},\mu^{⁎}))f(dist^{\times }(\mu^{⁎},\mu_{n+1})),$$

where:

$$M^{\times }(\mu_{n},\mu^{⁎})=\max\left\{\begin{array}{c} dist^{\times }(\mu_{n},\mu^{⁎}),\;dist^{\times }(\mu_{n},\mu_{n+1}),\;dist^{\times }(\mu^{⁎},\eta_{n}^{⁎}),\;\sqrt{dist^{\times }(\mu_{n},\eta_{n}^{⁎})dist^{\times }(\mu^{⁎},\mu_{n+1})} \end{array}\right\}.$$

Given n∈N, either one of the two following inequalities holds:

(6a) $$dist^{\times }(\mu^{⁎},\eta_{n}^{⁎})\leq \max\{dist^{\times }(\mu_{n},\mu^{⁎}),dist^{\times }(\mu_{n},\mu_{n+1}),dist^{\times }(\mu^{⁎},\mu_{n+1})\}$$

(6b) $$dist^{\times }(\mu_{n+1},\eta_{n}^{⁎})\leq \phi (dist^{\times }(\mu^{⁎},\eta_{n}^{⁎}))f\left(dist^{\times }(\mu^{⁎},\mu_{n+1})\right).$$

Suppose *n* satisfies (6b).

Letting $\theta_{n}:=\max\{dist^{\times }(\mu_{n},\mu^{⁎}),dist^{\times }(\mu_{n},\mu_{n+1}),dist^{\times }(\mu^{⁎},\mu_{n+1})\}$ one obtains:

(7) $$\frac{dist^{\times }(\mu^{⁎},\eta_{n}^{⁎})}{\phi (dist^{\times }(\mu^{⁎},\eta_{n}^{⁎}))f(dist^{\times }(\mu^{⁎},\mu_{n+1}))}\leq \frac{dist^{\times }(\mu^{⁎},\eta_{n}^{⁎})}{dist^{\times }(\mu_{n+1},\eta_{n}^{⁎})}\leq dist^{\times }(\mu^{⁎},\mu_{n+1})\leq \theta_{n}\;\frac{dist^{\times }(\mu^{⁎},\eta_{n}^{⁎})}{\phi (dist^{\times }(\mu^{⁎},\eta_{n}^{⁎}))}\leq \theta_{n}\cdot f(dist^{\times }(\mu^{⁎},\mu_{n+1}))$$

Equation (7) is still satisfied if *n* satisfies (6a). Thus, as n→∞, $\theta_{n}\to 1$, $f(dist^{\times }(\mu^{⁎},\mu_{n+1}))\to f(1)=1$ and $\frac{dist^{\times }(\mu^{⁎},\eta_{n}^{⁎})}{\phi (dist^{\times }(\mu^{⁎},\eta_{n}^{⁎}))}\to 1$. Thus $dist^{\times }(\mu^{⁎},\eta_{n}^{⁎})\to 1$ and $\eta_{n}^{⁎}\to \mu^{⁎}$. Since $T\mu^{⁎}$ is closed and $\{\eta_{n}^{⁎}\}\subset T\mu^{⁎}$, one obtains that $\mu^{⁎}\in T\mu^{⁎}$. □

Note that if in Theorem 3.6, we choose *f* and *ϕ* such that $f(\epsilon )=\epsilon^{\omega }$ and $\phi (\epsilon )=\epsilon^{\kappa }$, with ω>0 and κ∈[0,1), we obtain a contractive condition

$$|\begin{array}{c} \text{ for each }(\mu ,\nu )\in E(G),\text{ and for each }\xi \in T\mu ,\text{ there exists }\eta \in T\nu \text{ such that: }\\ \;\;\;\;\;\;\;\;\;dist^{\times }(\xi ,\eta )\leq {\left[M^{\times }(\mu ,\nu )\right]}^{\kappa }{\left[dist^{\times }(\nu ,\xi )\right]}^{\omega } \end{array}$$

which is weaker than (5) in Theorem 3.5.

One can also deduce the following equivalent theorem of Theorem 3.6 by applying the methodology employed in subsection 3.1.

Theorem 3.7 *Let* T:S→CL(S) *be a mapping defined on a complete metric space* (S,dist) *equipped with a graph* G=(V(G),E(G)) *, and such that:*

(i) *For all*(μ,ν)∈E(G)*and all*ξ∈Tμ*there exists*η∈Tν*such that*dist(ξ,η)≤ψ(M(μ,ν))+g(dist(ν,ξ))*, where*ψ∈Ψ*, M is as defined in*(2)*, and where*g:[0,∞)→[0,∞)*is a continuous function at* 0 *such that* g(0)=0*;*
(ii) *For some* $\mu_{0}\in S$ *and* $\mu_{1}\in Tu_{0}$ *,* $\left(\mu_{0},\mu_{1})\in E(G\right)$ *;*
(iii) *For any* ξ∈Tμ *and* η∈Tν *, if* dist(ξ,η)<dist(μ,ν) *then* (μ,ν)∈E(G)⇒(ξ,η)∈E(G) *;*
(iv) *For each sequence* $\{\mu_{n}\}\subset S$ *such that* $\left(\mu_{n},\mu_{n+1})\in E(G\right)$ *and* $\mu_{n}\to \mu$ *,* $\left(\mu_{n},\mu )\in E(G\right)$ *for each* n∈N *.*

*The map* T *has a fixed point.*

## 4 Numerical examples

Define on the set S=[0,∞), the multiplicative metric $dist^{\times }(\mu ,\nu )=e^{\left|\mu -\nu \right|}$.

Given α∈[0,1) and β∈[1,∞), define the map T:S→CL(S) by:

$$T\mu =\left\{ \begin{array}{cc} \left[0,\lambda (\mu )\right] & \text{ if }\;\mu <\beta ,\;\text{ where }\;\lambda (\mu )=\beta -1+e^{\alpha (\mu -\beta )}\\ \left\{\beta \right\} & \text{ if }\;\mu =\beta ,\\ \left[0,\gamma (\mu )\right] & \text{ otherwise,}\;\text{ where }\gamma :[\beta ,\infty )\to [0,\beta ]. \end{array}\right.$$

Note that the function λ:[0,β]→[0,β] is strictly increasing since its derivative is positive. Also, *λ* is *α*-Lipschitz. Indeed, from the Mean Value Theorem, if 0≤μ≤ν≤β, there exists $\theta_{\mu \nu }\in [\mu ,\nu ]$ such that $\lambda (\nu )-\lambda (\mu )=\lambda^{\prime }(\theta_{\mu \nu })(\nu -\mu )$, and $\lambda^{\prime }(\theta_{\mu \nu })=\alpha e^{\alpha (\theta_{\mu \nu }-\beta )}$ <*α*, hence λ(ν)−λ(μ)≤α(ν−μ).

Consider the graph G=(S,E(G)) with vertices the points of S and with E(G)={(μ,ν):0≤μ,ν≤β}∪{(μ,μ):μ∈S}. Given (μ,ν)∈E(G), either 0≤μ,ν≤β or μ=ν. Thus, four cases can be obtained:

Case 1: Suppose 0≤μ,ν<β. Then Tμ=[0,λ(μ)] and Tν=[0,λ(ν)].

Let ξ∈Tμ.

If μ≤ν then taking η=ξ∈Tμ⊂Tν, one obtains $dist^{\times }(\xi ,\eta )=1\leq {\left[dist^{\times }(\mu ,\nu )\right]}^{\alpha }$.

Suppose now that μ>ν.

If ξ∈Tν, then taking η=ξ∈Tν, $dist^{\times }(\xi ,\eta )=1\leq {\left[dist^{\times }(\mu ,\nu )\right]}^{\alpha }$; if ξ∈Tμ∖Tν, then taking η=λ(ν), $dist^{\times }(\xi ,\eta )=e^{\left|\xi -\eta \right|}=e^{\xi -\lambda (\nu )}\leq e^{\lambda (\mu )-\lambda (\nu )}={\left[dist^{\times }(\mu ,\nu )\right]}^{\alpha }$.

Therefore, for all ξ∈Tμ, there is η∈Tν such that $dist^{\times }(\xi ,\eta )\leq {\left[dist^{\times }(\mu ,\nu )\right]}^{\alpha }$.

In fact, for all ξ∈Tμ and η∈Tν such that $dist^{\times }(\xi ,\eta )<dist^{\times }(\mu ,\nu )$, since λ(μ)<β and λ(ν)<β, then 0⩽ξ,η<β, hence (ξ,η)∈E(G).

Case 2: Suppose 0≤μ<β and ν=β. Then Tμ=[0,λ(μ)] and Tν={β}.

Let ξ∈Tμ; taking η=β∈Tν, $dist^{\times }(\xi ,\eta )=e^{\left|\xi -\eta \right|}=e^{\left|\xi -\beta \right|}=dist^{\times }(\nu ,\xi )$.

Therefore, for all ξ∈Tμ, there is η∈Tν such that $dist^{\times }(\xi ,\eta )\leq dist^{\times }(\nu ,\xi )$.

In fact, for all ξ∈Tμ and η∈Tν be such that $dist^{\times }(\xi ,\eta )<dist^{\times }(\mu ,\nu )$, since λ(μ)<β and η=β, then 0≤ξ<η=β, hence (ξ,η)∈E(G).

Case 3: Suppose 0≤ν<β and μ=β. Then Tμ={β} and Tν=[0,λ(ν)].

Let ξ∈Tμ. Then ξ=β. Taking η=λ(ν)∈Tν, $dist^{\times }(\xi ,\eta )=e^{\beta -\lambda (\nu )}<e^{\beta -\nu }=dist^{\times }(\nu ,\xi )$.

Therefore, for all ξ∈Tμ there exists η∈Tν such that $dist^{\times }(\xi ,\eta )\leq dist^{\times }(\nu ,\xi )$.

Case 4: Suppose μ=ν.

For all ξ∈Tμ, taking η=ξ∈Tμ=Tν, we have that $dist^{\times }(\xi ,\eta )=1\leq {\left[dist^{\times }(\mu ,\nu )\right]}^{\alpha }$.

Therefore, for all ξ∈Tμ there exists η∈Tν such that $dist^{\times }(\xi ,\eta )\leq {\left[dist^{\times }(\mu ,\nu )\right]}^{\alpha }$.

In fact, if μ=ν≤β then for all ξ∈Tμ and η∈Tν, 0≤ξ,η≤β and (ξ,η)∈E(G), and if μ=ν>β, then for all ξ∈Tμ and η∈Tν, ξ≤γ(μ)≤β and η≤γ(ν)≤β so (ξ,η)∈E(G).

In all cases, for each ξ∈Tμ, there is η∈Tν such that either $dist^{\times }(\xi ,\eta )\leq {\left[dist^{\times }(\mu ,\nu )\right]}^{\alpha }$ or $dist^{\times }(\xi ,\eta )\leq dist^{\times }(\nu ,\xi )$.

This proves that for each (μ,ν)∈E(G) and ξ∈Tμ, there is η∈Tν satisfying the condition $dist^{\times }(\xi ,\eta )\leq \phi (M^{\times }(\mu ,\nu ))f\left(dist^{\times }(\nu ,\xi )\right)$, where $\phi (\epsilon )=\epsilon^{\alpha }$ and f(ϵ)=ϵ. Thus (*i*) of Theorem 3.6 is satisfied.

We also proved that for each (μ,ν)∈E(G), each ξ∈Tμ, and each η∈Tν, the pair (ξ,η) is an edge, hence (iii) of Theorem 3.6 is also satisfied.

Let $\left\{\mu_{n}\right\}$ be a sequence of points in S such that $\left(\mu_{n},\mu_{n+1})\in E(G\right)$ and $\mu_{n}\to \mu$.

If $0\leq \mu_{n},\mu_{n+1}\leq \beta$ for all n∈N∪{0}, then 0≤μ≤β since [0,β] is closed in S and so $0\leq \mu_{n}$, μ≤β and $\left(\mu_{n},\mu )\in E(G\right)$.

Suppose now that there is a non-negative integer $n_{0}$ such that $\mu_{n_{0}}>\beta$. Then $\left(\mu_{n_{0}},\mu_{n_{0}+1})\in E(G\right)$ ⇒ $\mu_{n_{0}+1}=\mu_{n_{0}}>\beta$ and by induction, $\mu_{n}=\mu_{n_{0}}>\beta$ for all $n\geq n_{0}$, and $\mu =\mu_{n_{0}}>\beta$. Also, $\left(\mu_{n_{0}-1},\mu_{n_{0}})\in E(G\right)$ ⇒ $\mu_{n_{0}-1}=\mu_{n_{0}}$ and recursively, $\mu_{n}=\mu_{n_{0}}$ for all $n\leq n_{0}$. Thus, $\mu_{n}=\mu_{n_{0}}=\mu$ for all non-negative integer *n* and $\left(\mu_{n},\mu )\in E(G\right)$ for all non-negative integer *n*. Hence (iv) of Theorem 3.6 is satisfied.

Condition (ii) is satisfied for $\mu_{0}=\mu_{1}=\beta$. In fact, any point μ∈[0,β] is a fixed point of T, an indication that the fixed point of a map satisfying the conditions of Theorem 3.6 may not be unique.

The following example, similar to the previous, is that of a map whose fixed point is unique, and which can be proven in a similar fashion to satisfy conditions (i)−(iv) of Theorem 3.6. From the proof of Theorem 3.6, uniqueness enables us to approximate the fixed point of a map satisfying the conditions of Theorem 3.6 via any sequence $\{\mu_{n}\}\subset S$ such that $\mu_{n+1}\in T\mu_{n}$ and $\left(\mu_{n},\mu_{n+1})\in E(G\right)$ for each n≥0.

Example 4.1 For α∈[0,1) and β∈[1,∞), define on the multiplicative metric space S=[0,∞) (with $dist^{\times }(\mu ,\nu )=e^{\left|\mu -\nu \right|}$) the map $T_{\alpha ,\beta }:S\to \mathrm{CL}(S)$ by:

$$T_{\alpha ,\beta }(\mu )=\left\{ \begin{array}{cc} \left[\lambda (\mu ),\beta \right] & \text{ if }\;\mu <\beta ,\;\text{ where }\;\lambda (\mu )=\beta -1+e^{\alpha (\mu -\beta )}\\ \left\{\beta \right\} & \text{ if }\;\mu =\beta ,\\ \left[0,\gamma (\mu )\right] & \text{ otherwise,}\;\text{ where }\gamma :[\beta ,\infty )\to [0,\beta ]. \end{array}\right.$$

$T_{\alpha ,\beta }$ has a fixed point as it satisfies conditions (i)−(iv) of Theorem 3.6, with the graph G=(S,E(G)) where E(G)={(μ,ν):0≤μ,ν≤β}∪{(μ,μ):μ∈S}. In fact, μ=β is the unique fixed point of $T_{\alpha ,\beta }$. Using MATLAB, we obtain convergence to the fixed point *β* of the sequence ${\left\{\mu_{n}\right\}}_{n\in N\cup \{0\}}$ defined by $\mu_{0}=0$ and $\mu_{n}=\lambda (\mu_{n-1})$, n∈N (see Table 1, Table 2). Such a sequence $\left\{\mu_{n}\right\}$ is such that any pair $\left(\mu_{n},\mu_{n+1}\right)$ of consecutive terms is an edge.

**Table 1:** When *α* = 0.5 and *β* = 2.

*n*	*μ*_*n*_
0	0
1	1.3679
2	1.7290
3	1.8733
4	1.9386
5	1.9698
6	1.9850
7	1.9925
8	1.9963
9	1.9981
10	1.9991
11	1.9995
12	1.9998
13	1.9999
14	1.9999
15	2.0000

**Table 2:** When *α* = 0.9 and *β* = 4.

*n*	*μ*_*n*_
0	0
1	3.0273
2	3.4167
3	3.5916
4	3.6924
5	3.7582
6	3.8044
7	3.8386
8	3.8648
9	3.8854
10	3.9020
11	3.9156
⋮	⋮
29	3.9904
⋮	⋮
50	3.9990

## Declarations

### Author contribution statement

Ige, Aminat O.; Olaoluwa, Hallowed O.; Olaleru, Johnson O.: Conceived and designed the experiments; Performed the experiments; Analyzed and interpreted the data; Contributed reagents, materials, analysis tools or data; Wrote the paper.

### Funding statement

This research did not receive any specific grant from funding agencies in the public, commercial, or not-for-profit sectors.

### Data availability statement

No data was used for the research described in the article.

### Declaration of interests statement

The authors declare no conflict of interest.

### Additional information

No additional information is available for this paper.
